# Supplementary material for: The interactome of intact mitochondria by cross-linking mass spectrometry provides evidence for coexisting respiratory supercomplexes
Source: Mol Cell Proteomics. 2017 Dec 8;17(2):216–32. doi: 10.1074/mcp.RA117.000470 (PMC5795388; doi:10.1074/mcp.RA117.000470)
Supplement: Supplemental Data [file supp_RA117.000470_133922_0_supp_23978_fzffwf.zip › spectra_annotation/mito_DR_spectra_annotation/77-1-4-1-4-1.pdf]

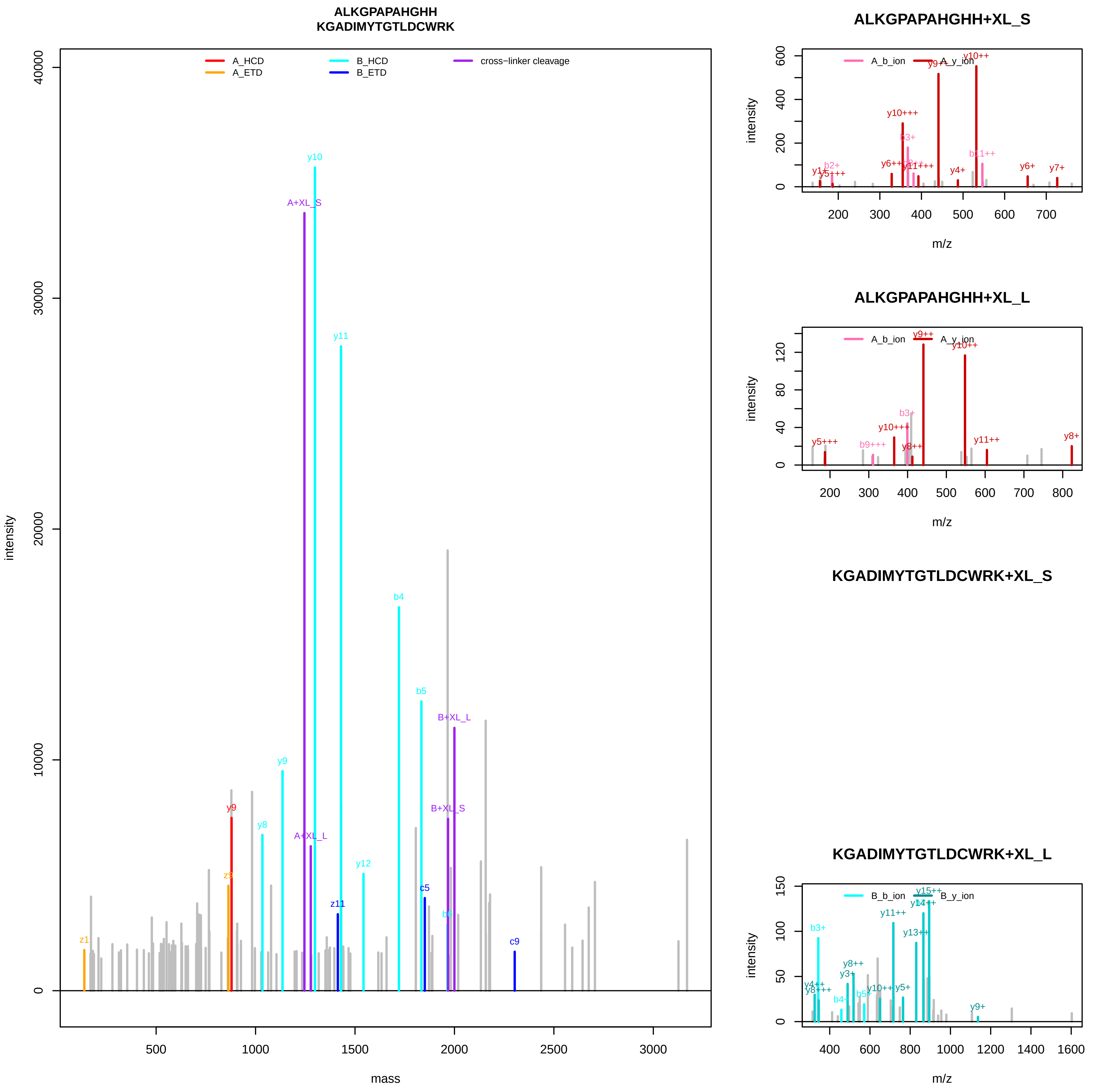

ALKGPAPAHGHH
KGADIMYTGTLDCWRK
A_HCD
A_ETD
B_HCD
B_ETD
cross−linker cleavage
intensity
mass
0
10000
20000
30000
40000
500
1000
1500
2000
2500
3000
z1
z9
y9
y8
y9
A+XL_S
A+XL_L
y10
z11
y11
y12
b4
b5
c5
b6
B+XL_S
B+XL_L
c9
ALKGPAPAHGHH+XL_S
A_b_ion
A_y_ion
y1+
b2+
y5+++
y6++
y10+++
b3+
y9++
y11+++
y10++
y4+
b11++
y6+
y7+
m/z
ALKGPAPAHGHH+XL_L
y5+++
b9+++
y10+++
b3+
y8++
y9++
y10++
y11++
y8+
KGADIMYTGTLDCWRK+XL_S
KGADIMYTGTLDCWRK+XL_L
B_b_ion
B_y_ion
b3+
y4++
y8+++
y3+
y8++
b4+
b5+
y10++
y5+
y11++
y13++
y14++
y15++
y9+
